# Evaluation of the Immunomodulatory Effects of Radiation for Chimeric Antigen Receptor T Cell Therapy in Glioblastoma Multiforme

**DOI:** 10.3390/cells13131075

**Published:** 2024-06-21

**Authors:** David Akhavan, Siddharth Subham, John D. Jeppson, Brenda Aguilar, Robyn A. Wong, Jonathan C. Hibbard, Susanta Hui, Jeffrey Y. C. Wong, Stephen J. Forman, Darya Alizadeh, Christine E. Brown

**Affiliations:** 1 Department of Radiation Oncology, University of Kansas Cancer Center, Kansas City, KS 66160, USA; dakhavan@kumc.edu (D.A.); s905s741@kumc.edu (S.S.); jjeppson@kumc.edu (J.D.J.); 2 Department of Hematologic Malignancies and Cellular Therapeutics, University of Kansas Cancer Center, Kansas City, KS 66160, USA; 3 Department of Cancer Biology, University of Kansas Cancer Center, Kansas City, KS 66160, USA; 4 Bioengineering Program, University of Kansas, Lawrence, KS 66045, USA; 5 Department of Immuno-Oncology, City of Hope Beckman Research Institute, Duarte, CA 91010, USA; baguilar@coh.org (B.A.); robwong@coh.org (R.A.W.); jhibbard@coh.org (J.C.H.); sforman@coh.org (S.J.F.); dalizadeh@coh.org (D.A.); 6 Department of Hematology and Hematopoietic Cell Transplantation, City of Hope National Medical Center, Duarte, CA 91010, USA; 7 Department of Radiation Oncology, City of Hope National Medical Center, Duarte, CA 91010, USA; shui@coh.org (S.H.); jwong@coh.org (J.Y.C.W.)

**Keywords:** glioblastoma, CAR T cell therapy, radiation, IL13Rα2, c-GAS-STING pathway, tumor microenvironment

## Abstract

Standard-of-care treatment for Glioblastoma Multiforme (GBM) is comprised of surgery and adjuvant chemoradiation. Chimeric Antigen Receptor (CAR) T cell therapy has demonstrated disease-modifying activity in GBM and holds great promise. Radiation, a standard-of-care treatment for GBM, has well-known immunomodulatory properties and may overcome the immunosuppressive tumor microenvironment (TME); however, radiation dose optimization and integration with CAR T cell therapy is not well defined. Murine immunocompetent models of GBM were treated with titrated doses of stereotactic radiosurgery (SRS) of 5, 10, and 20 Gray (Gy), and the TME was analyzed using Nanostring. A conditioning dose of 10 Gy was determined based on tumor growth kinetics and gene expression changes in the TME. We demonstrate that a conditioning dose of 10 Gy activates innate and adaptive immune cells in the TME. Mice treated with 10 Gy in combination with mCAR T cells demonstrated enhanced antitumor activity and superior memory responses to rechallenge with IL13Rα2-positive tumors. Furthermore, 10 Gy plus mCAR T cells also protected against IL13Rα2-negative tumors through a mechanism that was, in part, c-GAS-STING pathway-dependent. Together, these findings support combination conditioning with low-dose 10 Gy radiation in combination with mCAR T cells as a therapeutic strategy for GBM.

## 1. Introduction

Glioblastoma (GBM) is the most common primary malignant brain tumor in adults. Despite decades of research, this diagnosis is uniformly fatal, with a median overall survival of approximately 15–19 months after standard-of-care management [1]. Radiation is a standard-of-care approach for GBM and results in a modest improvement in overall survival [2]. However, clinical studies optimizing the radiation dose and fractionation alone have not resulted in improved long-term survival [3,4].

In addition to a direct tumor cytotoxic effect, radiation has also been described to immunomodulate the tumor microenvironment (TME) [5]. Radiation can lead to the promotion of antitumor immune responses, both locally and systemically, by various mechanisms [5], and a growing body of research supports evaluation with combination immunotherapies. Radiation-induced tumor cell death has been shown to result in the release of cytokines, chemokines, danger-associated molecular patterns (DAMPs), and tumor antigens [6]. These signals promote the infiltration of dendritic cells (DCs), cytotoxic T cells, and natural killer (NK) cells into the TME and lymph nodes [7,8]. Radiation-induced migration of APCs to the lymph nodes and the priming of effector T cells has been shown to result in systemic immune responses in preclinical studies [7]. The radiation-mediated upregulation of MHC molecules on tumor cells increases cytotoxic T cell activity by the presentation of new antigens [9], which could promote host antitumor immune responses.

The majority of preclinical studies have focused on radiation in combination with the checkpoint blockade [10], resulting in enhanced antitumor efficacy and overall survival. However, clinical trials in GBM evaluating the checkpoint blockade alone [11,12,13] or in combination with radiation [14,15] have failed to result in improved overall survival. The lack of efficacy/response observed in this combination therapy warrants additional investigation and further identification of the potential mechanism(s) of resistance.

Beyond checkpoint inhibitors, adoptive cell therapy holds promise as an effective therapeutic option for GBM. Chimeric Antigen Receptor (CAR) T cells combine the MHC-independent tumor recognition of the tumor-targeting domain (i.e., antibody or receptor ligand) with the cytolytic potency of a T cell. The first promising results of CAR T cell therapy were seen in hematological cancers. CD19-specific CAR T cells have shown efficacy in the treatment of many B cell malignancies, including refractory B-ALL and large B cell lymphoma, resulting in FDA approval [16,17], but the clinical success of CAR T therapy in solid tumors, including in GBM [18,19,20,21], has been more challenging. Our team was the first to clinically translate CAR T cells for the treatment of GBM. We have completed a phase I clinical trial (NCT02208362) that suggests the local delivery of IL13Rα2-targeted CAR T cells into the central nervous system (CNS) is safe and feasible, with evidence of bioactivity in some patients [19,22]. Additional clinical studies focused on malignant brain tumors have also reported safety as well as some bioactivity [18,20,21]. Interestingly, our clinical [19] and preclinical [23] experience of IL13Rα2-CAR T cells against GBM demonstrated that a strong CAR T antitumor response is associated with the activation of the host immune cells, suggesting that the host immune cells may play an important role in a successful CAR T therapy. Therefore, it is of interest to identify combination therapies that synergize with CAR T cell therapy by preconditioning the host immune response. Our team is currently evaluating the combination of the immune checkpoint blockade in a neoadjuvant or adjuvant setting with IL13Rα2-CAR T therapy for the treatment of recurrent GBM patients (NCT04003649). Radiation is also being explored as a valuable tool in modulating the immune response, and may augment CAR T therapy by addressing some of the barriers, such as tumor antigen heterogeneity and T cell trafficking [24]. The aim of this study was to leverage a murine immunocompetent model of GBM and CAR T cells to (1) identify the optimum neoadjuvant radiation dose that conditions the TME, (2) evaluate the potential synergy of combining condition radiation with CAR T therapy, and (3) evaluate the potential mechanism(s) that play a role in the efficacy observed in combining radiation and CAR T therapy. Utilizing an immunocompetent murine GBM model, we identify a conditioning stereotactic radiosurgery (SRS) dose that synergizes with CAR T therapy. We further evaluate the changes in glioma TME post-treatment and elucidate the potential mechanism(s) involved in the observed combination therapy.

## 2. Materials and Methods

**Mice and Cell Lines:** C57BL/6J parental and STING KO (C57BL/6J-Sting1^gt^/J) mice were purchased from Jackson labs (strain # 000664 and 017537, respectively). All mouse experiments were approved by the City of Hope Institutional Animal Care and Use Committee (IACUC). Mice were monitored for survival and symptoms related to tumor progression by the Center for Comparative Medicine at City of Hope, with euthanasia applied according to the American Veterinary Medical Association Guidelines. KR158B cells (a gift from Karlyne Reilly lab) [25] were transduced with firefly luciferase and murine IL13Rα2 (IL13Rα2^+^ KLuc). This cell line was maintained in DMEM (Gibco, Waltham, MA, USA) supplemented with 10% FBS (Hyclone Laboratories, Logan, UT, USA), 25 mmol/L HEPES (Irvine Scientific, Santa Ana, CA, USA), and 2 mmol/L L-glutamine (Lonza, Basel, Switzerland). Cell surface expression of mIL13Rα2 was authenticated by flow cytometry, and the cells were tested for Mycoplasma and maintained in culture for less than 3 months.

**Murine IL13Rα2-CAR T cell Production:** The murine IL13Rα2 CAR was constructed as previously reported [23]. Murine T cells were generated as previously described [23]. Briefly, T cells were isolated from the spleens of naïve C57Bl/6J mice with the EasySep Mouse T cell Isolation Kit (STEMCELL Technologies, Vancouver, Canada) and stimulated with Dynabead Mouse T-Activator CD3/CD28 beads (Gibco) at a 1:1 ratio. T cells were transduced on RetroNectin-coated plates (Takara Bio, San Jose, CA, USA) using a retrovirus-containing supernatant by performing spinoculation at 1500× *g* for 1 h at 32 °C with no break. Non-transduced and retrovirus-transduced T cells were next expanded for 4 days in RPMI 1640 (Lonza) supplemented with 10% FBS (Hyclone Laboratories), 55 mmol/L 2-mercaptoethanol (GIBCO), 50 U/mL recombinant IL2 (Novartis, Basel, Switzerland), and 10 ng/mL recombinant IL7 (Peptrotech, Cranbury, NJ, USA). Before in vivo experiments, the beads were magnetically separated from the T cells and the CAR T expression was determined by flow cytometry. Mock and CAR T cells were washed and resuspended in PBS before administering them into the mice.

**Radiation Dose Assessment:** We first identified the subtherapeutic dose of stereotactic radiosurgery (SRS) for our model by using increasing doses of single-fraction irradiation (0, 5, 10, and 20 Gy) utilizing the X-Rad precision radiation machine targeting the skull burr hole by a Cone Beam CT scan, under anesthesia, 6 days after the intracranial implantation of 1 × 10^5^ mIL13Rα2^+^ KLuc tumor cells. Tumor growth kinetics were measured using bioluminescence imaging (BLI). The BLI of the tumor burden was measured with SPECTRAL LagoX (Spectral Instruments Imaging, Tucson, AZ, USA) and analyzed using Aura software (v2.3.1, Spectral Instruments Imaging). We also performed RNA tissue analysis (Nanostring) 2 days after the SRS treatment (day 8) (refer to Nanostring section in Materials and Methods).

**Combination SRA and mCAR T Therapy**: After identifying an appropriate subtherapeutic radiation dose, we tested the combination of focal radiation with locally delivered IL13Rα2 CAR T cells. In brief, C57/BL6 mice were orthotopically injected with IL13Rα2^+^ KLuc. An orthotopic tumor model was established by the stereotactic injection of 1 × 10^5^ tumor cells intracranially (i.c.) into the right forebrain of 8-to-10-week-old C57BL/6J mice. Engraftment was verified by BLI. The mice were randomized into groups based on the BLI signal. The mice then underwent a dose of 10 Gy on day 6 and/or 0.5 × 10^6^ IL13Rα2CAR T cell injection at the tumor site on day 8. BLI of the tumor burden was measured with the SPECTRAL LagoX (Spectral Instruments Imaging) and analyzed using Aura software (v2.3.1, Spectral Instruments Imaging). Kaplan–Meier survival curves were generated by GraphPad Prism Software (v8).

For the tumor rechallenge experiments, the clearance of the tumor (in mice previously treated by IL13Rα2 CAR T cells or a combination of 10 Gy + IL13Rα2CAR T cells) was verified by BLI prior to orthotopic tumor rechallenge in the contralateral brain, where the mice were injected with 5 × 10^4^ mIL13Rα2^+^ KLuc or parent (mIL13Rα2^−^) cells in C57BL/6J wild-type or STING KO mice, and followed the tumor growth and survival.

**Nanostring:** Brains from euthanized mice were removed at the indicated time points, and a rodent brain matrix was used to cut along the coronal and sagittal planes to obtain a 4 mm × 4 mm section, centered around the injection site. These sections were then placed in Trizol. The RNA was purified using the miRNeasy mini-Kit (Qiagen, Hilden, Germany), following the manufacturer’s instructions (Qiagen). The RNA samples were subsequently quantified using a Nanodrop 1000 Spectrophotometer (Thermo Fisher Scientific, Waltham, MA, USA) and Qubit 3.0 Fluorometer (Thermo Scientific). The RNA fragmentation and quality control were determined by 2100 Bioanalyzer (Agilent, Santa Clara, CA, USA) assays. All samples were normalized to 20 ng/uL with the use of RNAse-free distilled water, and 100 ng/well of RNA was applied for the subsequent Nanostring analysis.

Samples were analyzed using the nCounter mouse PanCancer Immune profiling gene expression panel (NanoString Technologies, Seattle, WA, USA), as follows: 100 ng of RNA was first hybridized with Codeset from the gene panel at 65 °C for 16 h. The post-hybridization probe–target mixture was then processed with an automated nCounter Prep Station and quantified with an optical nCounter Digital Analyzer, and all data analysis was performed on nSolver (NanoString Technologies).

Normalization was performed by using the geometric mean of the positive control counts as well as the normalization genes present in the CodeSet Content. Gene expression analysis was performed using the nSolver v3.0 and Advanced analysis module software v2.0.134 (Nanostring Technologies).

**Statistics:** Comparisons across groups were made using pairwise Student *t*-tests after appropriate normality checks when the outcomes were continuous and using chi-square tests when discrete. Survival data were analyzed using Kaplan–Meier methods and log-rank tests to check the statistical significance of the survival curves. Analyses were carried out using either GraphPad Prism software (v5) or R (v4.0.2). No adjustments were made for multiple testing.

## 3. Results

### 3.1. Conditioning SRS Has Immunomodulatory Effects in Murine GBM

**Figure 1:**
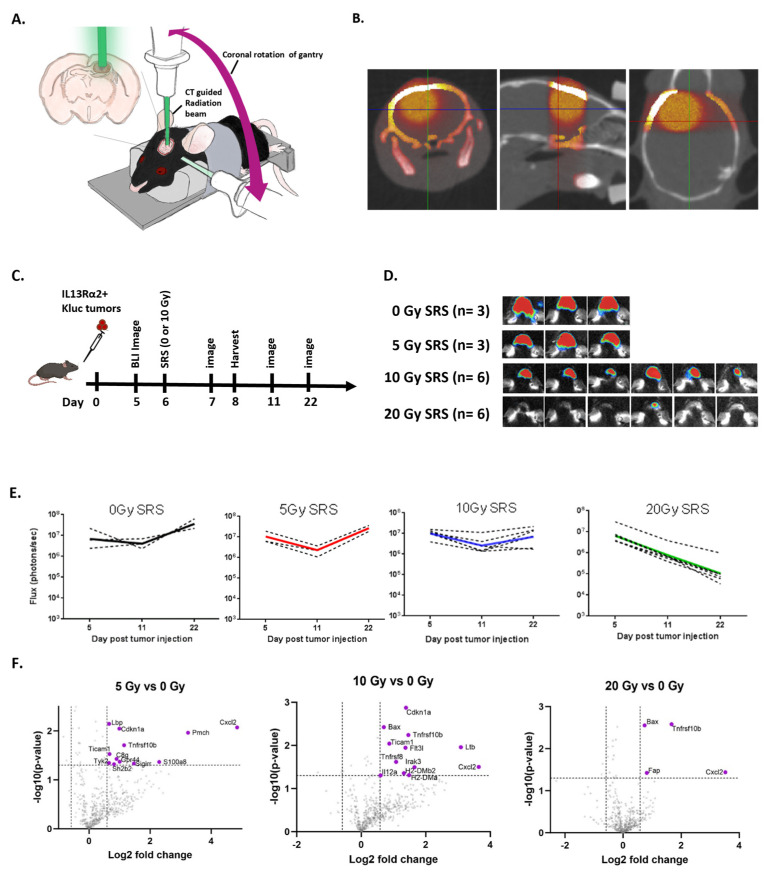
Changes in tumor growth and tumor microenvironment after dose-escalated stereotactic radiosurgery (SRS). (**A**) Mouse stereotactic radiosurgery (SRS) platform targeting the tumor injection site. (**B**) The 10 Gy SRS dose color wash in coronal, sagittal, and axial slices. (**C**) Schema of the experimental design. Mice bearing KR158B-mIL13Rα2^+^ gliomas that received different doses of radiation (0, 5, 10, or 20 Gy) 6 days after tumor implantation. Bioluminescent (BLI) images were taken before and after radiation. On day 8, tumor samples were harvested for the Nanostring analysis and the remaining mice were monitored for tumor growth. (**D**) Representative BLI images from day 22 for each treatment group is shown. (**E**) Graphs showing the tumor growth kinetics in the mice given titrating doses of radiation (0, 5, 10, or 20 Gy). (**F**) Nanostring analysis of tumors harvested at day 8, 2 days after the exposure to different radiation doses (0, 5, 10, or 20 Gy).

We first assessed the impact of titrated single-fraction radiation doses on the GBM TME, since this is clinically feasible in the recurrent GBM setting after having received full-course chemoradiation [26,27]. For these studies, we used the KR158 [25] syngeneic immunocompetent murine GBM model, which recapitulates the highly invasive GBM [25]. This tumor line is derived from a spontaneous glioma arising from *Nf1* and *Trp53* mutant mice, and is poorly immunogenic, as indicated by its unresponsiveness to anti-PD-1 checkpoint therapy [28]. Using the X-Rad smART precision radiation platform, SRS was applied locally at titrated doses of 0, 5, 10, and 20 Gy in mice bearing orthotopic intracranial KR158 tumors expressing murine IL13Rα2 and firefly luciferase (IL13Rα2^+^ KLuc) (Figure 1A–C). These doses of radiation were assessed by tumor growth kinetics using bioluminescent imaging (BLI) (Figure 1D,E) and changes in gene expression patterns in the TME (Figure 1F). The treatment of established IL13Rα2^+^ KLuc tumors (day 7) with 20 Gy focal radiation showed a strong cytotoxic effect, with tumor reduction observed in all of the mice by BLI. By comparison, doses of 5 Gy and 10 Gy exhibited negligible therapeutic effects. All three radiation groups (5, 10, and 20 Gy) showed some degree of immunomodulatory effects compared to the nontreated mice as assessed by the Nanostring RNA tissue analysis (nCounter^®^) 2 days post-SRS. For example, all three radiation doses showed a strong upregulation of the chemo-attractant molecule CXCL2 compared to the untreated controls (Figure 1F). Moreover, both 10 and 20 Gy upregulated tumor necrosis factor (TNF)-related genes (*Tnfrsf10b* and *Tnfrsf8* after 10 Gy and *TNfrsf10b* after 20 Gy). Only the 10 Gy dose induced the upregulation of class II histocompatibility antigen-related genes (*H2-DMb2* and *H2-DMa*), as well as the upregulation of *FMS-like tyrosine kinase 3* and the *FLT3* ligand, which is involved in dendritic cell development and maintenance (Figure 1F). Additionally, only the 10 Gy dose was associated with the upregulation of *IL12a*, a pro-inflammatory cytokine produced by antigen-presenting cells (APCs) which activates natural killer (NK) cells and induces the differentiation of naïve CD4 + T cells to become interferon-gamma (IFN-γ)-producing T helper 1 (Th1) effectors [29]. The 20 Gy-treated group had an overall paucity of gene changes compared to the other groups, which may be due to the more cytotoxic radiation dose (Figure 1F). Based on these findings, we next evaluated the impact of 10 Gy SRS in combination with mCAR T cell therapy, since this intermediate dose of radiation showed more favorable immunomodulatory effects without altering the tumor growth kinetics.

### 3.2. Conditioning SRS in Combination with mCAR T Therapy Enhances Antitumor Responses

We next evaluated the effect of combining radiation and CAR T cell therapy for antitumor efficacy, utilizing our previously established immunocompetent mouse model of the IL13Rα2-CAR T-cell platform [23]. Briefly, as previously reported [19], the murine IL13Rα2-CAR T (mCAR T) consists of the murine IL13 tumor-targeting domain, murine CD8 hinge (mCD8h), murine CD8 transmembrane domain (mCD8tm), murine 4-1BB costimulatory domain (m4–1BB), and murine CD3 zeta (mCD3ζ) [23]. A T2A skip sequence separates the CAR from a truncated murine CD19 (mCD19t) used for cell tracking [23].

**Figure 2:**
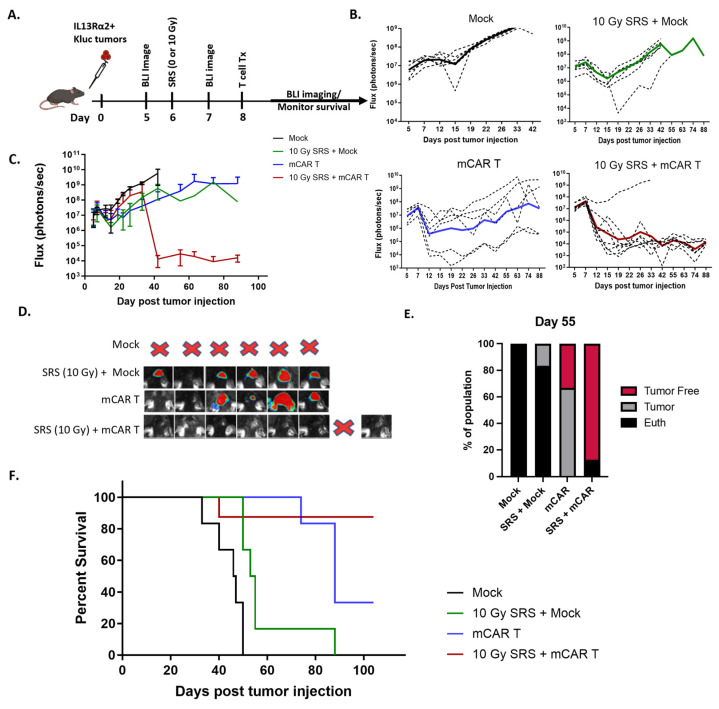
Conditioning SRS in combination with mCAR T treatment results in long-term tumor eradication. (**A**) Schematic of the experimental design; mIL13Rα2^+^ Kluc glioma cells (1 × 10^5^) were implanted orthotopically followed by a dose of 0 or 10 Gy on day 6 and mock or mCAR T (5 × 10^5^) intra-tumoral injection on day 8. (**B**) Graphs showing the tumor growth kinetics measured by BLI. (**C**) Graph showing the average flux (photon/s) over time in each treated group (day 88 comparison of mCAR T vs. 10GySRS+ mCAR T; *p* = 0.02). (**D**) Representative BLI images at day 55 for the animals that survived. A total of 6 animals per group, except for the combination therapy group that was n = 8. (**E**) Graph showing the number of tumor-free, tumor-bearing, and euthanized mice in each treatment group at day 55. (**F**) Kaplan–Meier graph showing the overall survival of each treatment group (mCAR T vs. 10GySRS+ mCAR T; *p* = 0.07).

For these studies, the mice underwent orthotopic intracranial implantation of mIL13Rα2^+^ Kluc, followed by BLI to confirm the tumor engraftment (Figure 2).

The mice were then treated with either conditioning 10 Gy alone (day 6 post-tumor injection), mCAR T cells alone (day 8), or both 10 Gy (day 6) followed by mCAR T cells (day 8). Tumor kinetics indicated negligible tumor regression in the mock plus 10 Gy SRS group as compared to mock-treated-alone group. By comparison, the combination of 10 Gy SRS plus mCAR T-treated groups significantly improved in antitumor activity compared to mCAR T-alone group (*p* = 0.02) (Figure 2B,C). The representative BLI of individual mice from day 55 further confirms that the majority of mice treated with SRS plus mCAR T showed tumor eradication (Figure 2D). Further, the number of animals surviving at 55 days was much higher in the combination treatment compared to mCAR T alone (7/8 vs. 2/6) (Figure 2E). Each of the three treatment conditions resulted in improved survival compared to the control mock-treated group (*p* < 0.005) (Figure 2F and Figure S1). Moreover, the mCAR T and the combination therapy were superior to the SRS plus mock therapy, *p* = 0.009 and *p* = 0.003, respectively. There was also a trend in the improved overall survival for the mice receiving the combination of radiation and mCAR as compared to mCAR alone (*p* = 0.07). Together, these findings support the therapeutic benefit for preconditioning the TME with radiation for CAR T cell therapy in the setting of GBM.

**Figure 3:**
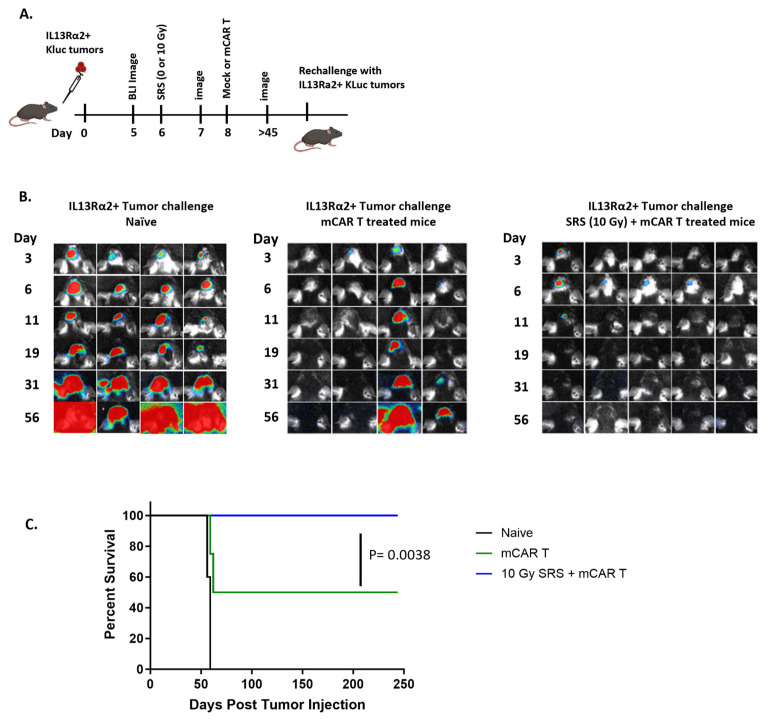
Conditioning SRS and mCAR T-treated mice reject tumor rechallenge. (**A**) Schematic of the experimental design; survived mice from the mCAR T or SRS (10 Gy) + mCAR T-treated groups were rechallenged with mIL13Rα2^+^ Kluc cells (5 × 10^4^). (**B**) BLI images demonstrating the tumor burden in naïve, mCAR T, and SRS (10 Gy) + mCAR T survived mice after rechallenge. (**C**) Kaplan–Meier graph showing the overall survival after the tumor rechallenge.

We have previously shown that mCAR T cell therapy can induce immunological memory against IL13Rα2-negative tumors [23]. To investigate the possibility that conditioning radiation might enhance mCAR T therapy in tumor rejection after rechallenge, the mice that survived from the mCAR T therapy or combination SRS plus mCAR T were rechallenged with mIl13Rα2-positive tumors in the contralateral brain. In this setting, 50% of the mice from the mCAR T-alone group rejected the tumor while, strikingly, all of the mice from the dual treatment had complete tumor rejection and long-term survival (*p* = 0.0038) (Figure 3). These results suggest that, while the mCAR T-treated group demonstrated antitumor response, the SRS plus mCAR T-treated group exhibited a more profound “recall” antitumor response.

### 3.3. Conditioning SRS and mCAR T Therapy Enhance Innate and Adaptive Immunity Pathways Involving the cGAS-STING Pathway

**Figure 4:**
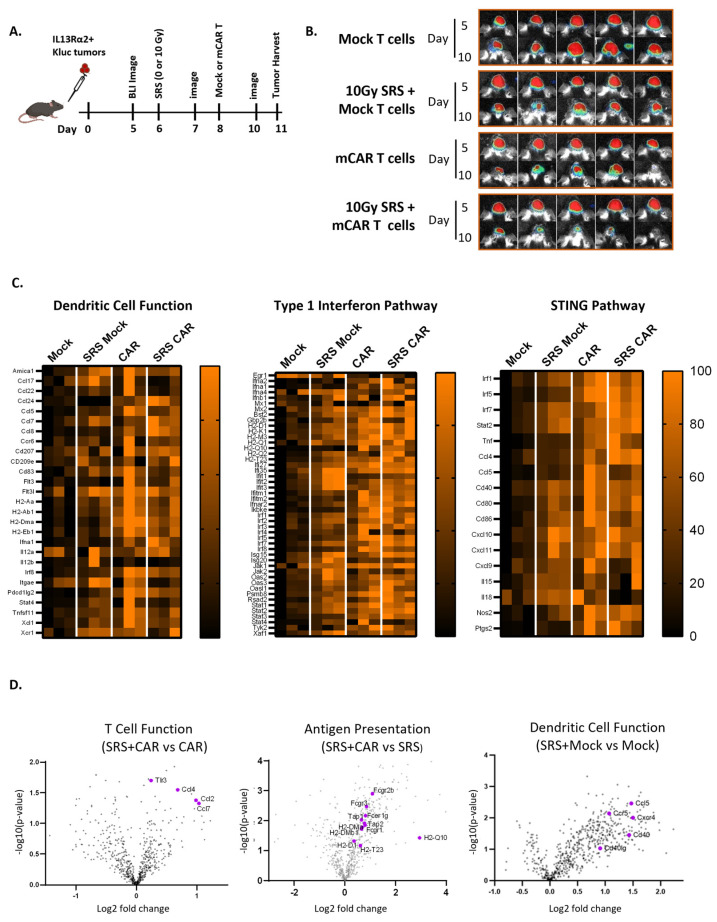
Gene expression analysis of tumor after combination conditioning SRS plus mCAR T treatment. (**A**) Schematic of the experimental design; mIL13Rα2^+^ Kluc glioma cells (1 × 10^5^) were implanted orthotopically, followed by 0 or SRS (10 Gy) on day 6 and mock or mCAR T (5 × 10^5^) intra-tumoral injection on day 8. Tumors were harvested 2 days after on day 11 and processed for Nanostring analysis. (**B**) BLI images showing the tumor burden on days 5 and 10. (**C**) Heat maps showing the changes in the expression of genes associated with the dendritic cell function (right), type 1 interferon (middle), and STING pathway (left). (**D**) Volcano plots identifying the specific genes upregulated in different treatment groups.

To reveal changes in the TME that are associated with the improved antitumor efficacy for the combination of conditioning SRS and mCAR T therapy, tumor/brain tissues were harvested from the mice after treatment with SRS (10 Gy) and/or mCAR T cells and processed for Nanostring RNA sequencing (nCounter^®^) (Figure 4A,B). Heat maps of the dendritic cells, type 1 IFN, and STING-related genes demonstrate enhanced gene expression in all three groups compared to the treatment with mock T cells (CAR-negative) (Figure 4C).

Treatment with mCAR T and combination SRS + mCAR T exhibited augmented global gene expression and genes associated with pathways in immune response compared to the SRS + mock group (Figure S2A). The combination of SRS plus mCAR T showed distinct gene expression patterns compared to mCAR T alone (the upregulation of the *TLR3* and *CCL* family) or compared to SRS (the upregulation of *H2-Q10*, *H2-T23*, *H2-DMb1*, and other MHC II-related genes). Nanostring gene enrichment scores were evaluated among the four different groups (mock, SRS + mock, mCAR, and SRS + mCAR T) in both innate and adaptive domains, and generally demonstrated upregulation compared to the mock treatment. For example, the domains of the MHC presentation, NK cell, interferon, antigen processing, innate immunity, TLR pathway, and complement pathway in all three groups showed statistically significant elevation compared to the mock treatment (Figure S2B). While there was no clear additive gene signature benefit to the combination therapy, these data highlight the role of both radiation and CAR T cells in activating innate and adaptive immune pathways important in the immune response.

The comparison of specific differences in gene expression patterns between the combination therapy and mCAR T alone identifies the increased expression of genes associated with T cell recruitment and function, including *Toll-Like Receptor 3* (*TLR3*) and *chemokine ligand (CCL) 2, 4*, and *7* in the combination group relative to the mCAR-alone group (Figure 4D; left panel).

Relative to the radiation alone, the combination group enhanced genes associated with antigen presentation, including *H2-Q10, H2-D1, H2-T23, H2-Dma, H2-DMb1, H2-T23*, and *Tap2* (Figure 4D; middle panel). Relative to the mock treatment, the SRS group demonstrated an increase in dendritic cell function genes such as *CD40, Cxcr4, Ccl5, Ccr5*, and *Cd40Ig* (Figure 4D; right panel). These data highlight the importance of radiation, as well as in combination with mCAR T, in the upregulation of MHC-II presentation, which is a well-characterized mechanism of dendritic cell recruitment and the induction of immunologic memory.

**Figure 5:**
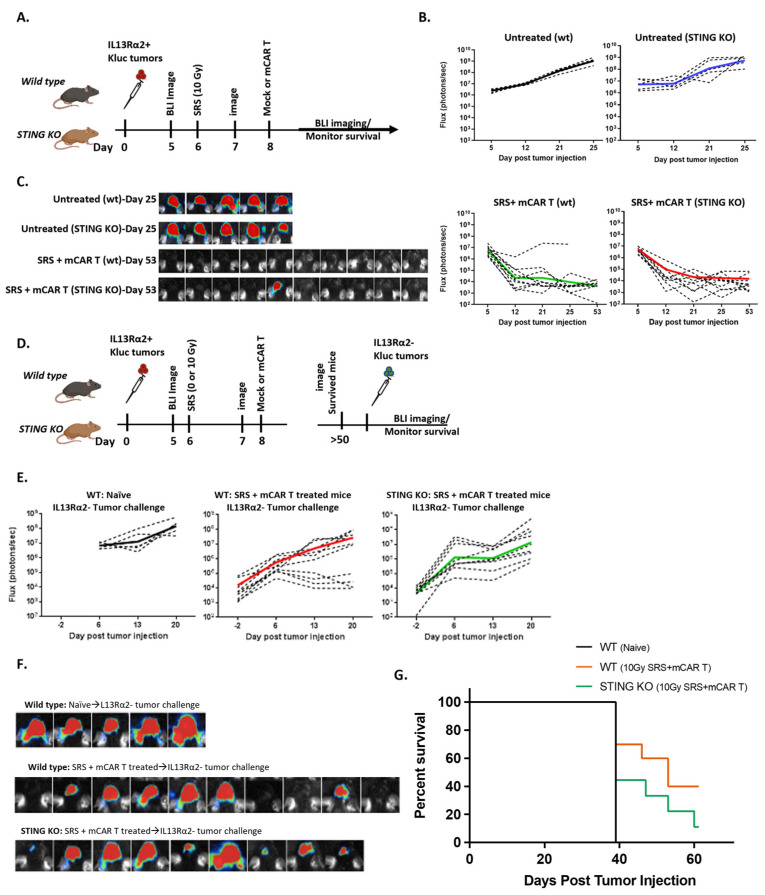
Induction of memory immune response is partly dependent on STING. (**A**) Schema of the experimental design. Wild-type (wt) and STING knockout (STING KO) mice were implanted with mIL13Rα2^+^ Kluc glioma cells (1 × 10^5^), then treated with SRS (10 Gy) + mCAR T (5 × 10^5^) and evaluated for tumor progression and overall survival. (**B**) Graphs showing tumor growth kinetics measured by BLI. (**C**) Representative BLI images from each group at day 25 or 53. (**D**) Schema of IL13Rα2-negative tumor challenge study. WT and STING KO mice that were treated with SRS (10 Gy) + mCAR T and survived were challenged with IL13Rα2-negative tumors (5 × 10^4^). (**E**) Graphs showing the tumor growth kinetics after the challenge with IL13Rα2-negative tumors in naïve (wt), SRS (10 Gy) + mCAR T-treated (wt), and SRS (10 Gy) + mCAR T-treated (STING KO) groups. (**F**) Representative BLI images from each group at day 20. (**G**) Kaplan–Meier graph showing the overall survival post-tumor challenge (10GySRS+ mCAR T wt vs. STING KO; *p* = 0.2).

The cyclic GMP-AMP synthase–stimulator of interferon genes (cGAS-STING), which results in type I interferon-dependent antitumor immunity, is an important pathway in radiation-mediated antitumor responses [30]. Given this important role of STING, as well as our observation of type interferon-1- and STING-related gene expression in our tumor model (Figure 4C), we sought to evaluate the role of host STING signaling in antitumor response observed after combination SRS plus mCAR T therapies. Wild-type (WT) and STING knockout (STING KO) mice were injected with IL13Ra2+ Kluc tumors. As previously described, the mice were treated with combination conditioning SRS (10 Gy) plus mCAR T (Figure 5A). Interestingly, potent antitumor responses were observed in both the WT and STING KO hosts that received the combination therapy regimen (Figure 5A–C), suggesting that STING pathway may not play a major role in initial antitumor activity.

Next, we sought to investigate whether there was a difference in the endogenous memory in the surviving mice from the WT vs. STING KO mice. When the surviving mice were rechallenged with a parent tumor line (IL13Rα2 negative), the STING KO mice demonstrated a modestly reduced antitumor response and survival benefit (Figure 5D–G). These results suggest that, while the STING pathway may not have impacted the initial CAR T-mediated antitumor responses observed post-therapies, it plays a role in the induction of memory immune response and the formation of immunologic memory against tumor cells.

## 4. Discussion

We previously reported that IL13Rα2-CAR T clinical and preclinical antitumor responses can be associated with the activation of the endogenous immune cells, which results in memory immune response against IL13Rα2-negative tumors [19,23]. Together, these findings suggest that recruiting and activating the host immune cells is important for a successful CAR T therapy in GBM.

Radiation is incorporated as an adjuvant treatment course in the management of GBM. The tumoricidal effects of radiation are well described in the management of GBM patients. Indeed, in a large, randomized study trial, radiation improved the overall survival compared to best conventional care from 14 to 35 weeks. Radiation potentiates cytotoxic effects mainly through DNA damage. Beyond this antitumor effect, radiation has a role in modulating the TME through multiple mechanisms, including the release of cytokines, chemokines, DAMPs, and tumor antigens [6].

With respect to GBM, syngeneic mouse models are an important tool to evaluate changes in the TME after radiation. Previous studies have evaluated changes in the TME after radiation in the syngeneic GBM lines GL261 and SB28 [31,32]. We chose to utilize the KR158B cell line, as it better recapitulates the hallmarks of GBM, including being highly invasive, myeloid-rich, and resistant to the checkpoint blockade [33,34]. Prior studies have evaluated radiation and temozolomide (TMZ) in KR158B but utilized whole-mouse brain radiation, which is not clinically relevant, as whole-brain radiation is neurotoxic [35]. The highly immunogenic GL261 model has previously been used to evaluate combination stereotactic radiation and the anti-PD-1 blockade, which demonstrated improved survival and increased tumor infiltration by cytotoxic T cells and decreased regulatory T cells in combination therapy [10]; unfortunately, to date, clinical trials evaluating the checkpoint blockade in combination with radiation in GBM have not demonstrated a survival benefit [36].

To better characterize the impact of SRS on the TME, we utilized the small-animal radiation research platform device (SARRP) that delivers radiation only at the tumor-implanted burr hole with stereotactic radiosurgery (SRS). Prior studies have only evaluated single-dose levels of stereotactic radiation or whole-brain radiation [24]. After assessing various doses of SRS, we report that relatively low doses of 10 Gy tumor irradiation upregulates the tumor necrosis factor receptor superfamily (*Tnfrsf10b* and *Tnfrsf8*) and *Ticam1*, which is involved in innate immunity, and adaptive immunity by *TLR3, TLR4* (through *TICAM2*), and *TLR5* to mediate NF-kappa-B and interferon-regulatory factor (IRF) activation and to induce apoptosis [37] (Figure 1). These results are consistent with other preclinical pancreatic cancer tumor models of low-dose radiation and CAR T cells enhancing antitumor responses, in part due to the tumor necrosis factor-related apoptosis-inducing ligand (TRAIL) [38]. Importantly, our data implicate other mechanisms of radiation-CAR T synergy beyond the TRAIL in a GBM model. Although much has been published on MHC class I upregulation after radiation [39], relatively little has been described regarding the radiation effects on MHC class II. Enhancing the antigen-presenting cell MHC class II-restricted tumor antigen presentation to CD4+ T cells is a critical issue for triggering protective immunity and re-orienting the TME toward an antitumor state [40]. Strikingly, we identified that a relatively low dose of 10 Gy upregulates the immune-modulating MHC class II gene (*H2-Dmb2* and *H2-Dma*) as compared to an ablative dose of 20 Gy (Figure 1). Importantly, the SRS plus mCAR T combination also increased MHC class II genes compared to radiation (Figure 4). Initial priming and triggering of naïve antigen-specific T helper cells are believed to be mediated by specialized MHC class II-positive DCs, which engulf antigen cell debris into peptides, and the presentation on MHC class II for T helper scrutiny. Further supporting this model, we also identified the gene signature of DC activation after SRS, which was also enhanced by the CAR T treatment (Figure S2). Our observation of increased MHC-II expression in the TME after SRS or SRS plus mCAR T, which is also supported in other murine solid tumor models, where a larger portion of the immunogenic mutanome is presented by MHC-II rather than MHC-I, resulting in recognition by CD4 T cells [41].

We also observed the upregulation of chemokine C-X-C motif 2 (gene *CXCL2*) after doses of 5, 10, and 20 Gy (Figure 1). CXCL2 is an inflammatory chemotactic agent produced by mast cells and macrophages, and which can recruit neutrophils [42]. Neutrophils have been implicated in both protumor and antitumor roles in the TME [43]. Tumor-associated neutrophils (TANs) have an antitumor N1 and protumor N2 subtype, similar to the classic M1 and M2 form of tumor-associated macrophages [44]. Indeed, *CXCL2* expression has been used to enhance the antitumor immunity of the oncolytic virus in orthotopic syngeneic murine breast cancer models through the enhancement of cytotoxic T lymphocytes [45]. Future studies will be required to further evaluate the role of *CXCL2* upregulation in augmenting the antitumor effect in our model.

Many challenges must be overcome to improve the effectiveness of immunotherapy in GBM. Multiple large, randomized studies have not shown any benefit to the immune checkpoint blockade in GBM. This is largely because GBM is devoid of tumor-infiltrating lymphocytes. De novo engineered T cells are currently under clinical study in GBM, including the targeting of the tumor-associated antigens IL13Rα2, HER2, and EGFRvIII (NCT04003649, NCT03696030, and NCT02664363) [18,21,22]. Early clinical results have demonstrated that the local delivery of CAR T into the CNS is safe and feasible, with some evidence of bioactivity, including one case report of complete regression after IL13Rα2 re-directed CAR T cells [19,22]. Immune correlative studies demonstrate host T cell infiltration after CAR T infusion followed by tumor recurrence in the setting of immuno-editing [19]. These data suggest the importance of the host immune system in the treatment response. Utilizing our immunocompetent murine CAR T model, and informed with clinical data of a unique patient responder to CAR T, we previously identified IFNγ as critical for CAR T cell-mediated myeloid activation and the induction of endogenous immunity [23]. Building upon this work, in this manuscript, we investigated how radiation may enhance CAR T treatment. Our data demonstrate mice cured with combination SRS plus mCAR T had improved antitumor response to mIL13Rα2^+^ Kluc rechallenge (Figure 2 and Figure 3). This supports our data of synergy with combination of SRS and mCAR T given the strong innate immunity and interferon gene signatures with either treatment (Figure 4). Our study builds on prior studies by Gambhir et al. [24], which demonstrated antitumor synergy with combination 5 Gy focal tumor radiation and anti-GD2 CAR T. Important differences remain, in that Gambhir et al. [24] injected CAR T cells intravenously, and tumor rechallenge was only reported after whole-mouse irradiation and not focal tumor radiation. In our study, CAR T cells were injected intracranially following focal tumor radiation, and we were able to demonstrate significant antitumor response after tumor rechallenge. To our knowledge, our study is the first to demonstrate focal tumor irradiation in combination with intracranial CAR T delivery resulting in long-term antitumor response and tumor rejection after rechallenge. Focal tumor irradiation as a preconditioning regimen is attractive because it may supplant more toxic regimens such as chemotherapy or total-body irradiation.

Based on our findings on the importance of IFNγ in CAR T-mediated tumor clearance and immune memory [23], as well as previously reported studies on the role of radiation in activation of type I interferon [46,47,48], we further delved into interferon-mediated immunologic memory mechanisms in our immunocompetent model. When irradiated cells release their contents, cGAMP synthase senses cytosolic DNA and activates a dendritic cell (DC)-mediated STING pathway. STING has been shown to be required for DC maturation and, in turn, the radiation-induced adaptive immune response in a murine model. Indeed, our Nanostring data implicate type I interferon, STING, and DCs in the SRS and CAR T treatment group (Figure 4 and Figure S2). To further confirm these findings, we next evaluated the importance of this pathway by using STING KO mice. Our results showed that the overall response to the combination SRS plus CAR T treatment was comparable in the STING KO and WT mice. To assess the immunological memory with respect to neoantigen spread beyond the mIL13Rα2 tumor antigen, mice were rechallenged with Kluc parental tumors (mIL13Rα2-negative). Here, the WT mice exhibited modestly improved memory responses compared to the STING KO host (Figure 5). These results suggest that different mechanisms exist for radiation-induced antitumor response vs. memory induction. These results highlight the role of the STING pathway in the induction of a host endogenous response, and support previously reported studies regarding STING agonists and radiation in multiple preclinical tumor models [49,50]. Together, our data implicate both innate and adaptive immune pathways for both radiation and CAR T cells that may, in concert, enhance the antitumor activity as well as the immunologic memory in a STING-dependent manner.

## 5. Conclusions

Here, we report that a successful strategy of low-dose 10 Gy SRS radiation followed by CAR T cells results in a long-term curative and antitumor response in the highly aggressive KR158B murine glioma model. Our results are clinically actionable, as IL13Rα2-targeting CAR T cells are currently being studied in clinical trials in recurrent GBM, where radiation is typically used to control tumor progression. This promising approach may be immediately implemented in patients as a preconditioning regimen prior to CAR T cell therapy against GBM.

## Data Availability

Data are available upon reasonable request. All data relevant to the study are included in the article or uploaded as supplementary information.

## Supplementary Materials

The following supporting information can be downloaded at: https://www.mdpi.com/article/10.3390/cells13131075/s1, Figure S1: The effect of conditioning SRS radiation plus mCAR T on tumor progression; Figure S2: Gene expression changes in different treatment groups.
