# Supplementary material for: Evaluation of the Immunomodulatory Effects of Radiation for Chimeric Antigen Receptor T Cell Therapy in Glioblastoma Multiforme
Source: Cells. 2024 Jun 21;13(13):1075. doi: 10.3390/cells13131075 (PMC11240512; doi:10.3390/cells13131075)
Supplement: Supplementary file 1 [file cells-13-01075-s001.zip › cells-3003316-supplementary.pdf]

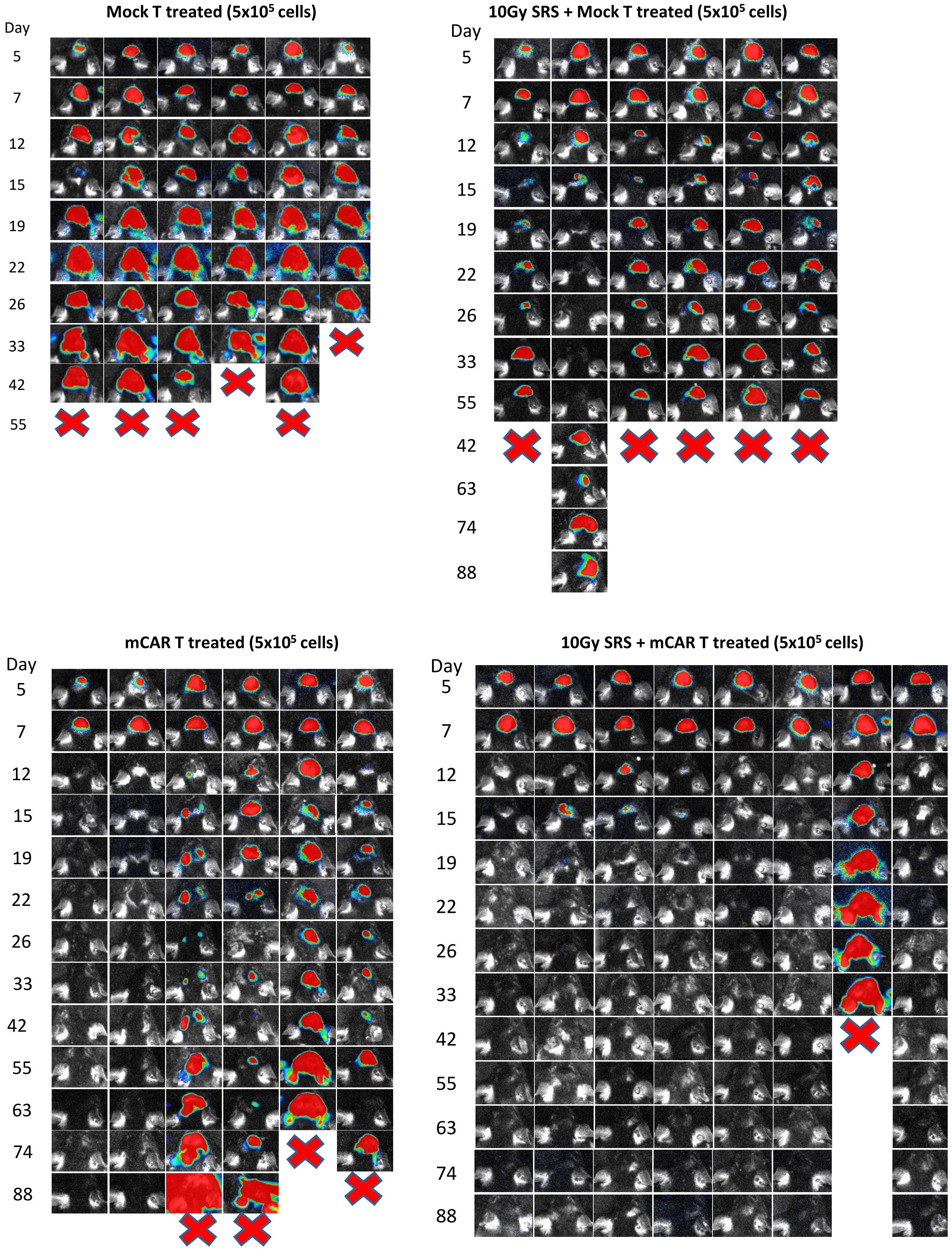

Supplemental Figure 1: The effect of conditioning SRS radiation plus mCAR T on tumor progression. BLI images of mice bearing mIL13Rα2+ Kluc glioma cells ($1x10^5$) before and treatment with Mock T, SRS (10 Gy) + Mock T ($5x10^5$), or SRS (10 Gy) + mCAR T ($5x10^5$).

A.

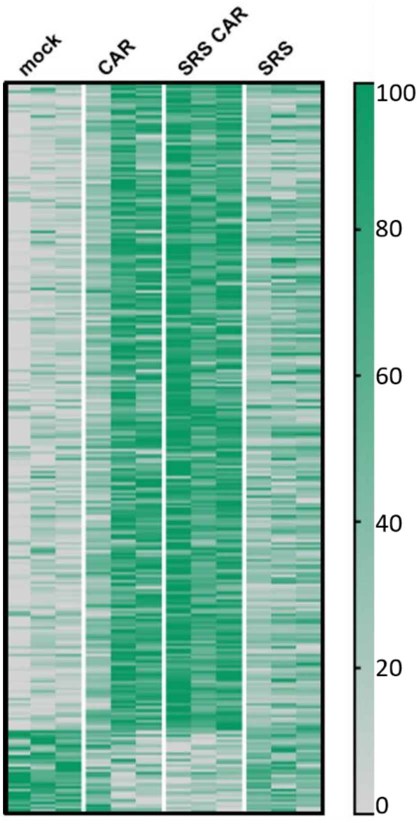

B.

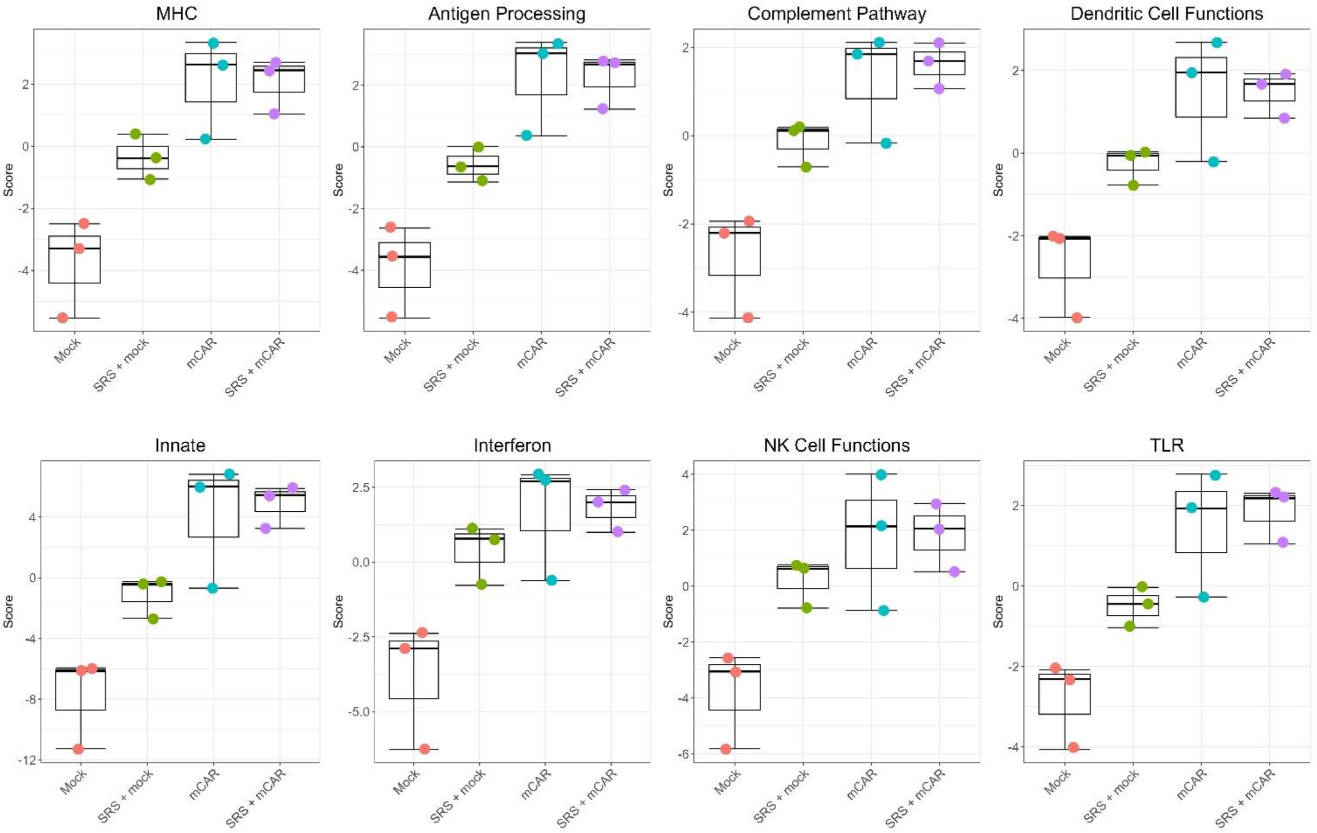

Supplemental Figure 2: Gene expression changes in different treatment groups. A. Heat map shows changes in expression of all genes. **B.** graphs demonstrate changes in gene signatures related to innate and adaptive immune system mCAR T, SRS (10 Gy) plus mock or SRS (10 Gy) plus mCAR T.
